# miRNA-seq analysis of high glucose induced osteoblasts provides insight into the mechanism underlying diabetic osteoporosis

**DOI:** 10.1038/s41598-024-64391-z

**Published:** 2024-06-11

**Authors:** Yang Zhang, Mengying Li, Pengqiang Lou, Minjie Zhang, Dan Shou, Peijian Tong

**Affiliations:** 1 The First Affilffiliated Hospital of Zhejiang Chinese Medical University (Zhejiang Provincial Hospital of Chinese Medicine), Institute of Orthopeadics and Traumatology, Hangzhou, China; 2 School of Pharmaceutical Sciences, Zhejiang Chinese Medical University, Hangzhou, China

**Keywords:** Diabetic osteoporosis, OGN, Osteoblasts, High glucose, Cell biology, Microbiology, Biomarkers, Diseases, Endocrinology, Pathogenesis

## Abstract

The present study aims to explore the etiology of Diabetic osteoporosis (DOP), a chronic complication associated with diabetes mellitus. Specifically, the research seeks to identify potential miRNA biomarkers of DOP and investigated role in regulating osteoblasts. To achieve this, an animal model of DOP was established through the administration of a high-sugar and high-fat diet, and then injection of streptozotocin. Bone microarchitecture and histopathology analysis were analyzed. Rat calvarial osteoblasts (ROBs) were stimulated with high glucose (HG). MiRNA profiles of the stimulated osteoblasts were compared to control osteoblasts using sequencing. Proliferation and mineralization abilities were assessed using MTT assay, alkaline phosphatase, and alizarin red staining. Expression levels of OGN, Runx2, and ALP were determined through qRT-PCR and Western blot. MiRNA-sequencing results revealed increased miRNA-702-5p levels. Luciferase reporter gene was utilized to study the correlation between miR-702-5p and OGN. High glucose impaired cell proliferation and mineralization in vitro by inhibiting OGN, Runx2, and ALP expressions. Interference with miR-702-5p decreased OGN, Runx2, and ALP levels, which were restored by OGN overexpression. Additionally, downregulation of OGN and Runx2 in DOP rat femurs was confirmed. Therefore, the miRNA-702-5p/OGN/Runx2 signaling axis may play a role in DOP, and could be diagnostic biomarker and therapeutic target for not only DOP but also other forms of osteoporosis.

## Introduction

Type 2 diabetes mellitus (T2DM) a prevalent hormonal metabolic disorder in humans, constituting over 90% of all individuals with diabetes. T2DM is characterized by high levels of blood glucose and urine glucose due to the deficiency and dysfunction of insulin metabolism. The presence of dyslipidemia, elevated blood glucose levels, and glucose in the urine may play a role in the onset of diabetic osteoporosis (DOP) and fractures of the bones^1^. Each year, there are over 90,000 osteoporotic fractures worldwide, with the majority linked to diabetic osteoporosis DOP and presenting a substantial risk to both human health and socioeconomic factors^2^. Disruption of glucolipid homeostasis in T2DM results in elevated levels of extracellular metabolites within the diabetic microenvironment. In recent years, thorough research on the diabetic microenvironment and mineral homeostasis has revealed that increased cortical porosity, imbalanced bone metabolism, and distorted bone microarchitecture are the primary features of DOP^3^. It is imperative for ongoing studies to focus on DOP, especially within a substantial cohort of T2DM patients. Growing evidence shows that T2DM patients have both deranged trabecular and cortical microarchitecture, which may increase the risk of fractures^4,5^. The associated dyslipidemia, hyperglycemia and glucosuria may contribute to the development of DOP and bone fractures. As a result, osteogenic differentiation may be affected due to changes in nutrition supplement, variations in hormones, and the loss of calcium. Because of the induced alterations in nutrition supplement, mutations in hormones and calcium loss. Therefore, more attention is needed to treat osteoporosis and prevent fractures in diabetic patients. In prior in vivo investigations, a T2DM rat model was established through the administration of a high-sugar and high-fat diet in conjunction with low-dose streptozotocin treatment. Subsequently, the T2DM animals exhibited diminished bone mass and decreased levels of bone turnover markers in comparison to the control group of rats^6,7^. However, the precise mechanism of DOP and relationship between high glucose and bone formation in vivo and in vitro are still unknown.

A plethora of studies suggest that miRNAs are endogenous, small (17–24 nucleotides), single-stranded and non-coding RNAs that regulate gene expression to affect various biological functions^8^. There has been an increase in interest in miRNAs among researchers recently and their target genes in bone formation and stimulation, regulating osteoblasts’ biological process^9^. For instance, miRNA-21 promoted the osteogenic ability of BMSCs by increasing P-Akt and HIF-1α activation^10^. MiRNA-34 was directly targeted Smad2 during osteoblast differentiation^11^. MiRNA-139-3p inhibited osteoblast differentiation and promoted osteoblast apoptosis^12^. To summarize, miRNA plays a crucial part in bone remodeling, encompassing both the differentiation and maturation of osteoblasts.

High glucose (HG) induced osteoblast was used to set up a model of diabetes-induced OP in vitro. Previous studies have shown that HG greatly decreased the viability of these cells^13^. Under conditions of high glucose, high-level blood glucose and advanced glycation end-products leads to excessive reactive oxygen species, and causes oxidative stress injury of osteoblasts^14^. However, the determination of particular miRNAs in osteoblasts induced by high glucose has not been achieved. Reliably quantifying miRNA levels is crucial for identifying new miRNAs and comprehending their impact. This can be accomplished by employing high throughput RNA sequencing technologies^15,16^.

In this study, we put forth the hypothesis that T2DM has an impact on the bone microarchitecture, leading to the development of osteoporosis. Additionally, we propose that the miRNA-702-5p/OGN/Runx2 signaling axis may serve as a potential pathway involved in the pathogenesis of diabetic osteoporosis. To investigate this, we established animal models of DOP and compared the bone microarchitecture differences between the control and model groups. Furthermore, we utilized high glucose-induced osteoblasts to validate the specific functionality of the candidate miRNA and target genes by using high-throughput sequencing of miRNA and mRNA. In a word, we hope the conclusions of our study will provide a new perspective on the pathogenesis and potential therapeutic targets of DOP.

## Materials and methods

### Animal modelling and experimental procedure

In total, 40 healthy Sprague–Dawley rats (male, 12 weeks old, body weight 200 ± 20 g) were acquired from the Zhejiang Academy of Traditional Chinese Medicine. The animal experiments protocols were approved by the Animal Ethics Committee of Zhejiang Academy of Traditional Chinese Medicine, and conformed to the ARRIVE guidelines (Approval No. [2022] 004). The rats were housed in a barrier at the Institution animal core of Zhejiang Academy of Traditional Chinese Medicine. The conditions for raising animals are as follows: a room with 23 ± 2 ℃ temperatures, 55 ± 5% humidity, and a 12 h light/dark cycle, besides, the tap water was freely used for animals, and the animals adapted to the new environment one week before the experiment. This study adhered to the ARRIVE guidelines (https://arriveguidelines.org) and all methods were conducted in accordance with applicable guidelines and regulations. Prior to sacrifice, the rats underwent a 12 h fasting period and were euthanized under mild sevoflurane-based anesthesia, following the American Veterinary Medical Association (AVMA) guidelines for anesthesia and euthanasia of animals.

The rats were divided into two groups (n = 20 in each group): the control group; and T2DM group (model group). The model group was fed a high-sugar and high-fat diet (Diet #MD 31.1% fat, 53.3% sugar, total calories 3.95 kcal/g; Mediscience Ltd, China) for 4 weeks. After 4 weeks, an STZ injection was administered intraperitoneally to rats (35 mg/kg body weight in 100 mL of sterile citrate buffer, pH 4.5) for two consecutive days. Following STZ administration, the blood glucose level was measured 72 h later. Fasting serum lisulin (FINS) levels were detected by ELISA method, following the protocols. A random selection of rats whose blood glucose levels exceeded the 16.7 mmol/L threshold was selected as diabetics for further study. The normal group was fed with conventional feed for the same time^17^.

### Bone microarchitecture and histopathology analysis

Each rat’s right femur was collected after sacrifice. The microarchitecture of the trabecular bone architecture was visualized on the distal femur in each treatment group, by using Micro-computed tomography (micro-CT, μCT-100 SCANCO Medical AG, Switzerland). The scan parameters were set at 70 kV voltage, 200 μA electric current, 15 μm resolution ratio, and 400 ms exposure time. Then, the software (Evaluation V6.5-3 Switzerland) was used to analyze the reconstructed images. As previously described, trabecular bone volume fraction (BV/TV), trabecular thickness (Tb.Th), bone surface density (BS/BV), trabecular separation (Tb.Sp), trabecular number (Tb.N), and bone mineral density (BMD) were measured for each sample.

Each rat’s right femur was collected after sacrifice, fixed in 4% (v/v) paraformaldehyde, embedded in paraffin, and decalcified with the decalcifying solution for one month. Colonic tissue was collected and fixed in 4% (v/v) paraformaldehyde, embedded in paraffin, and sliced into 5 μm. Then slides were stained with hematoxylin and eosin (H&E), according to the routine procedure, and then a microscopic examination (Olympus, Olympus-CX41, Japan) was performed, and the images were captured at 200 magnifications. The Percentage of trabecular ratios was calculated by measuring trabecular area compared to the total area of the section, using the ImageJ software.

### Immunohistochemistry (IHC) staining

The bone sections were analyzed by immunohistochemistry (IHC) staining to examine the expression of OGN and Runx2. In short, sections were treated with dewaxing, water merging, and antigen retrieval process. The sections were incubated with OGN (bs9407R, Bioss), OCN (bs-0470R) and Runx2 (bs-1134R, Bioss) antibodies at 4 ℃ overnight, and incubated with goat anti-rabbit IgG (1:200) at room temperature for 1 h. After the dyeing process, the slices are washed with PBS. A microscope (Olympus, Olympus-CX41, Japan) was used to observe the positive intensity of OGN, OCN, and Runx2. The images of IHC were captured at 100 and 200 magnifications.

### Osteoblasts culture and treatment

Rat calvarial osteoblasts (ROBs) were isolated from neonatal (2-day-old) Sprague-Dawley rats, as published previously^18^. Subsequently, cells were cultured in α-MEM (Sigma-Aldrich, Haverhill, UK) supplemented with 2 mmol/L L-glutamine, 100 U/mL penicillin, 100 mg/mL streptomycin and 10% fetal bovine serum (Sigma-Aldrich) at 37 °C with 5% CO_2_ until confluence was reached. Cells were trypsinized and plated in 6 well plates, the passage of four osteoblasts were used for all the following experiments. Glucose powder (Sigma-Aldrich) was dissolved in Phosphate buffered saline (PBS) to add to α-MEM. The ROBs were then treated with 10, 20, and 50 mg/mL of glucose and the same concentration of vehicle control.

Seeding ROBs in 6 well plates and growing them to 60–80% confluence. The cells were transfected with miR-702-5p mimics/ inhibitor (GenePharm Pharmaceutical Technology Co., Ltd., Shanghai, China), and OGN siRNA (GenePharm Pharmaceutical Technology Co., Ltd., Shanghai, China) for 6 h with Lipofectamine 2000 (Invitrogen, Waltham, MA, USA) or GP-transfect-Mate (GenePharm Pharmaceutical Technology Co., Ltd., Shanghai, China). Each transfection was performed in triplicate. After transfecting 24 and 48 h, cells were harvested.

### miRNA-seq of HG-induced ROBs

In order to explore differential expressions of miRNAs, ROBs were treated with HG (20 mg/mL) for 24 h. Total RNA of control and model groups was extracted using Trizol reagent (Invitrogen, USA). The quantity and purity were analyzed by Bioanalyzer 2100 (Agilent, USA) with RIN number > 7.0. Approximately 1 μg of total RNA was used to prepare a small RNA library according to a protocol of TruSeq Small RNA Sample Prep Kits (Illumina, USA). Illumina Hiseq 2500 (LC-BIO, Hangzhou, China) was used to perform miRNA-seq. The sequence quality was verified using FastQC.

### Quantitative real-time PCR verification of miRNAs and target genes

The sequence data of miRNA-702-5p was validated by qRT-PCR using a 7500 real-time PCR system (Applied Biosystems) with AceQ SYBR® Green Master Mix (Vazyme) according to the manufacturer’s instructions. The results of the experiments were normalized to the expression of the constitutive β-actin gene. The forward and reverse primers sequences were shown in Supplementary Table 1.

### Western blot analysis

After treatment with different concentration of glucose and transfecting with miR-702-5p mimic/ inhibitor and OGN siRNA, ROBs were washed with ice-cold PBS and total cell lysates were prepared using RIPA lysis buffer (SIGMA- ALDRICH®). After centrifugation at 10000 rpm for 20 min, the supernatant was collected. Protein concentration was quantified by using the BCA Protein Assay kit (KeyGEN BioTech Co., Ltd., Jiangsu, China). 20 μg proteins were subjected to 12% or 10% SDS-PAGE and the separated proteins were transferred to 0.45-µm PVDF membranes (Bio-Rad Co., Ltd., CA, USA). The protein bands were blocked with 5% BSA in PBST for 1 h, and then probed with primary antibodies, such as GAPDH (1:1000 dilution; Abcam), Runx2 (1:1000 dilution, Abcam), ALP (1:1000 dilution, Abcam,) and OGN (1:1000 dilution; Abcam) at 4 °C overnight. Anti-Rabbit-HRP conjugated secondary antibodies (1:2000 dilution; R&D Systems®) were used to detect the primary antibodies Then the PVDF membranes were visualized in the ChemiDocTM MP Imaging system (BioRad Co., Ltd., CA, USA) using a chemiluminescent ECL reagent (BioRad Co., Ltd., CA, USA).

### Dual-luciferase reporter assay

HEK 293 cells were seeded into 96-well plates. OGN 3′-UTR was amplified and cloned into the GPmiRGLO vector (GenePharm Pharmaceutical Technology Co., Ltd., Shanghai, China). Cells were co-transfected with 50 nM miRNA-702-5p or scrambled mimic and 400 ng of dual luciferase vector expressing the wild-type or mutant OGN. After 24 h, a luciferase reporter gene assay was performed using a dual-luciferase reporter assay kit (Yeasen Biotechnology Co., Ltd., Shanghai, China). The ratios of renilla luciferase activity to firefly luciferase activity were calculated to evaluate the target relationship of miRNA-702-5p and OGN.

### Cell proliferation assay and cell staining

MTT assay was used to measure the proliferation rates of osteoblasts. Osteoblasts were dispensed into 96-well plates, and at the end of the 24 h culture, a total of 50 μL of MTT solution was put into the cell and incubated at 37° C for 4 h. Then, the solution was discarded, and DMSO solution was added to each well. At last, the absorbance was measured at 570 nm.

ALP staining was performed following the ALP staining kit protocols (KGI Biotechnology Co., Ltd., Jiangsu, China). The osteoblasts were cultured in a calcification induction medium for 14 days. After induction, cells were fixed in 4% formaldehyde and washed with PBS, and stained with 0.1% alizarin red. The orange or red nodes were identified as calcium nodules.

### Statistical analysis

The data were expressed as mean ± standard error. T-test and Wilcox’s test were used to analyze the difference between the two groups. For other distribution and statistical comparisons, analysis of each group was performed using the analysis of variance (ANOVA) test and the least significant difference (LSD) test in statistical Product and service solutions analysis software. The analysis probability value with P less than 0.05 was considered statistically significant.

## Results

### T2DM decreased the parameters of the trabecular bone’s microarchitecture

**Figure 1:**
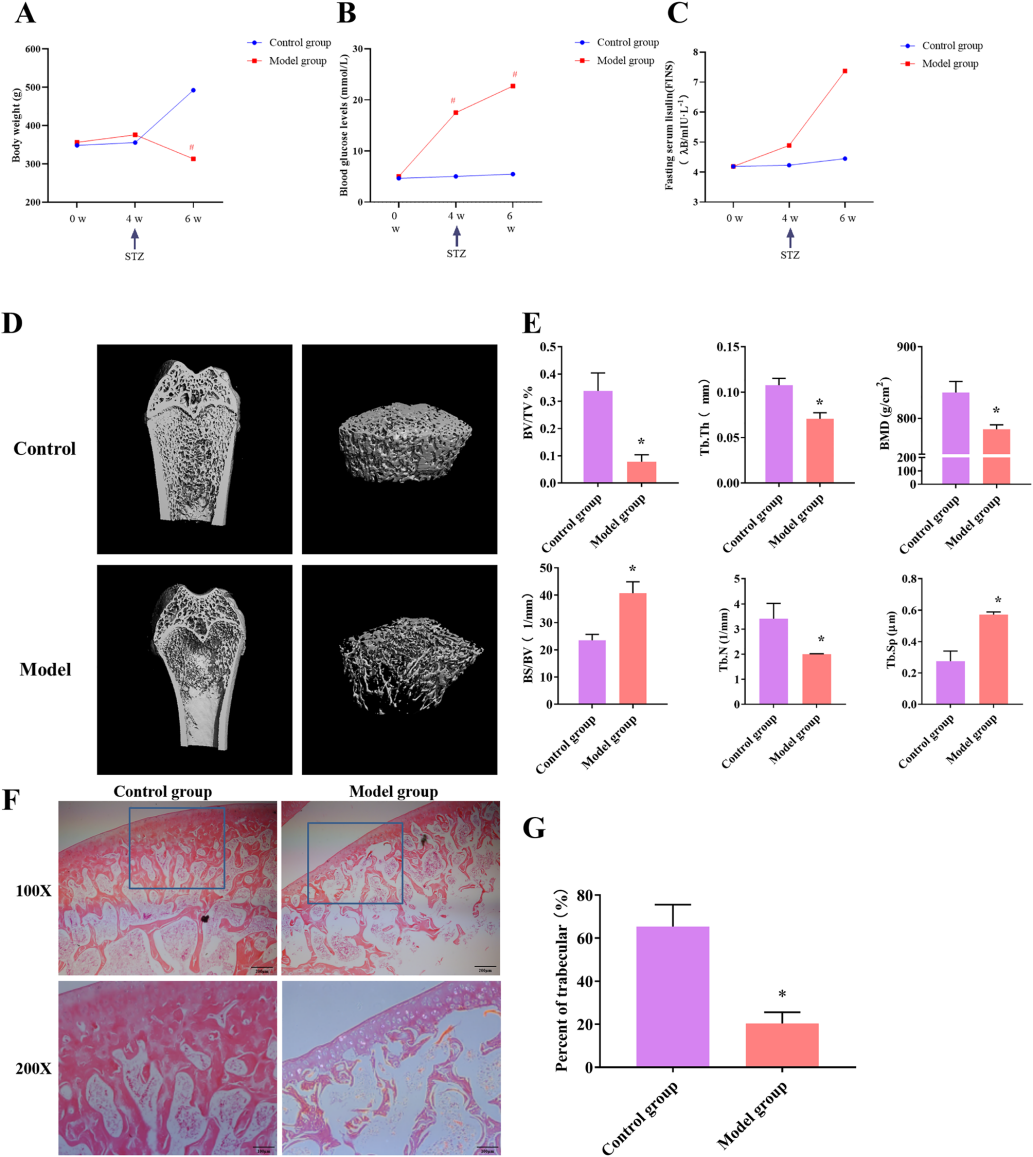
The three-dimensional micro-CT images of the femur and histopathology analysis in each group. (**A**–**C**) Body weights and blood glucose levels, and fasting serum lisulin (FINS) levelsof animals in each group. *P < 0.05, compared with the control groups. (**D**) Three-dimensional reconstruction of bone defects of the femur 8 weeks after modeling. (**E**) The results of microarchitecture results of femur. Data depict the mean ± standard deviation (mean ± SD) and are representative of three independent experiments. *P < 0.05, compared with the control groups. (**F**) H&E staining images of femur in each group (× 200, scale bar: 100 μm). (**G**) The percent of trabecular in femurs. Data depict the mean ± standard deviation (mean ± SD) and are representative of three independent experiments. *P < 0.05, compared with the control groups.

In the T2DM model group, rats’ body weights were reduced significantly than the control group from weeks 4 to 6, which showed the main features of diabetic weight loss. (Fig. 1A). Meanwhile, the blood glucose levels of the rats in the model group were higher than the control group after the high-sugar and high-fat diet feeding for 4 weeks and STZ injection (Fig. 1B). Furthermore, the FINS levels of the rats in the experimental group exhibited an increase following a 4-week high-sugar and high-fat diet, and further elevated after STZ injection, consistent with the development of insulin resistance in T2DM. In comparison with the control group, the bone trabecular in the model group were severely damaged, with broken phenomena, disordered arrangement, and obvious separation. And the bone shape is severely deformed and the compact bone substance bent inward and the bone cavity became smaller (*P* < 0.05) (Fig. 1D). DOP significantly decreased femur morphology and mineral density.

The micro-CT and 3D reconstruction results (Fig. 1E) showed lower BMD, BV/TV, Tb.Th, and Tb.N, higher BS/BV and Tb.Sp values at the distal femur in the model groups than that in the control group, as well as the breakage of cancellous bone at the distal metaphysis of the femur. The microarchitecture of trabecular bone was changed in T2DM model that induced osteoporosis.

The femurs in each group were subjected to H&E staining to observe the histological changes. H&E staining revealed the destruction of femoral trabeculae and decreased amounts of trabecular tissue at the distal metaphysis of the femur in all the diabetic group compared to the control groups (Fig. 1F, G).

### Identiffcation and validation of differentially expressed miRNAs in HG-induced ROBs

**Figure 2:**
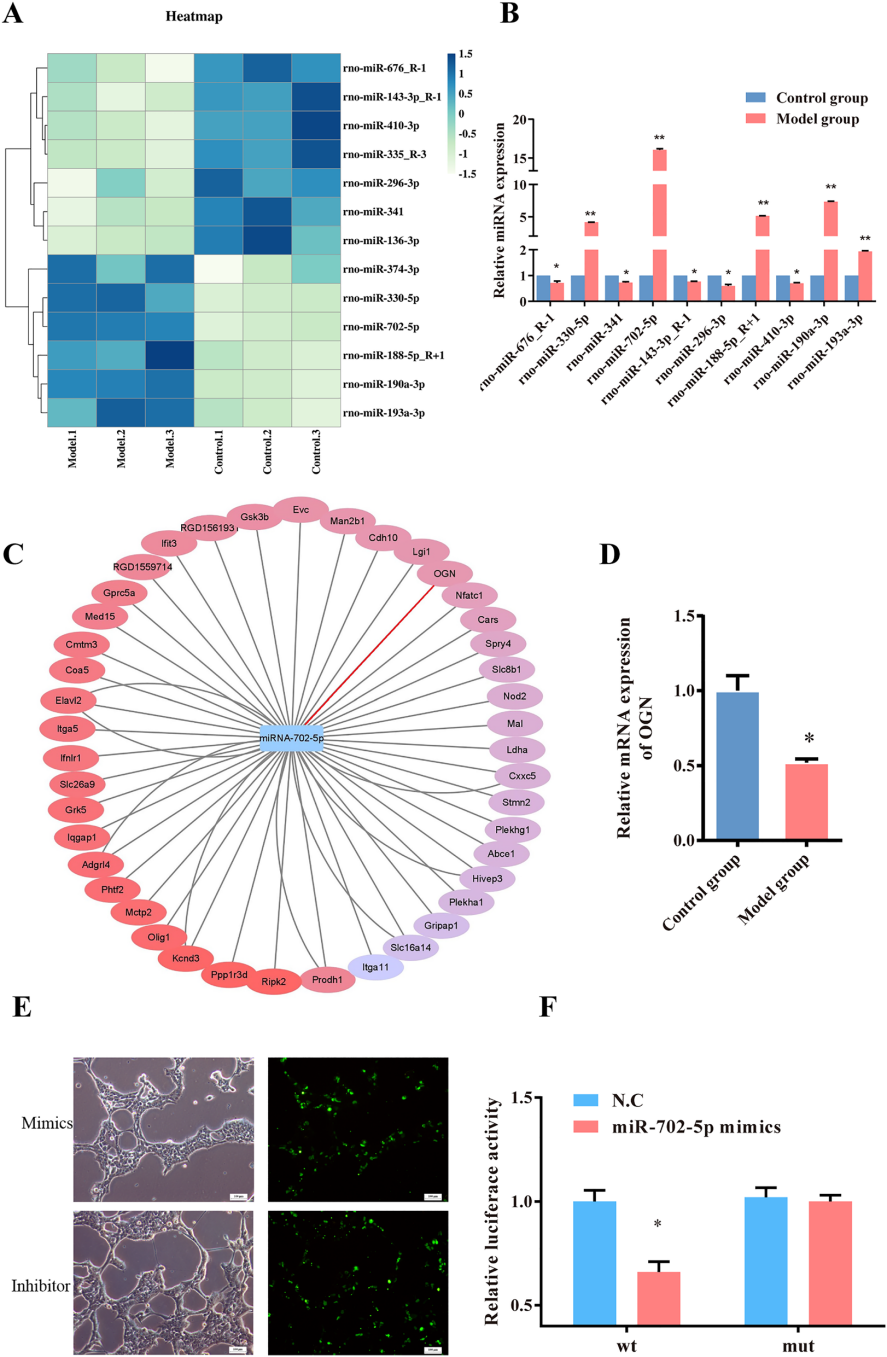
MiRNA-seq results in HG induced ROBs and verification of MiRNA-702-5p directly targets OGN. (**A**) Heatmap of differential expressions of top 10 differential expressed miRNAs between HG induced ROBs (model groups) and control groups. (**B**) RT-PCR verification results of the 10 differential expression miRNAs, and miRNA-702-5p was significantly up regulated. **P < 0.001, *P < 0.05, compared to the control groups. (**C**) The network analysis was performed by the Cytoscape 6.1 software to indicate the relationships between the miRNA-702-5p and the target genes. (**D**) RT-PCR verification results of the target gene expression of OGN, *P < 0.05, compared to the control groups. (**E**) The transfection efficiency of mimics and inhibitor in HEK293 was approximately higher than 80%. (**F**) Interaction between miRNA-702-5p and OGN 3’-UTR tested in the luciferase reporter assays. *P < 0.05, compared to the N.C groups.

HG-induced ROBs and control groups were subjected to next-generation microRNA analysis. The raw data of miRNA sequencing (miRNA-seq) of high glucose induced osteoblasts were uploaded to NCBI (BioProject ID: PRJNA918841). The miRNA-seq results indicated there were 10 miRNAs differential expressed (*P* < 0.05) between the model and control groups (Fig. 2A, Table 1). Then, the differential expressed miRNAs were detected in the model (HG-induced ROBs) and control groups by qRT-PCR. Among them, miR-702-5p, was shown to exhibit sharp rise in model groups, by verifying the top ten differential expression miRNAs (Fig. 2B). The network analysis was performed by the Cytoscape 6.1 software to indicate the relationships between the miR-702-5p and the target genes (Fig. 2C). OGN is related to bone development and was detected in the target genes of miR-702-5p. The mRNA expression of OGN was significant lower in HG-induced ROBs than that in the control group (Fig. 2D). The assays with luciferase reporter assays demonstrate that miRNA-702-5p reduces OGN transcription in HEK 293. When cells were co-transfected with OGN wt and miRNA-702-5p mimics, luciferase activity was suppressed (*P* < 0.05). However, no suppression was seen when cells were co-transfected with OGN mut or miRNA-702-5p mimics (Fig. 2 E, F).

**Table 1:** The differentially expressed miRNAs assed by RNA-Seq.

miRNA	miRNA sequence	*p*-value	Trend
rno-miR-676_R-1	CCGTCCTGAGCTTGTCGAGCT	9.32E-03	Down
rno-miR-330-5p	TCTCTGGGCCTGTGTCTTAGGC	1.19E-02	Up
rno-miR-341	TCGGTCGATCGGTCGGTCGGT	1.29E-02	Down
rno-miR-702-5p	GTGAGTGGGGTGGTTGGCATG	1.31E-02	Up
rno-miR-143-3p_R-1	TGAGATGAAGCACTGTAGCTC	1.37E-02	Down
rno-miR-296-3p	GAGGGTTGGGTGGAGGCTCTCC	2.16E-02	Down
rno-miR-188-5p_R + 1	CATCCCTTGCATGGTGGAGGGC	3.25E-02	Up
rno-miR-410-3p	AATATAACACAGATGGCCTGT	3.25E-02	Down
rno-miR-190a-3p	ACTATATATCAAGCATATTCCT	3.34E-02	Up
rno-miR-193a-3p	AACTGGCCTACAAAGTCCCAGT	3.89E-02	Up

### MiR-702-5p targeted OGN inhibit the osteoblast’s activity

**Figure 3:**
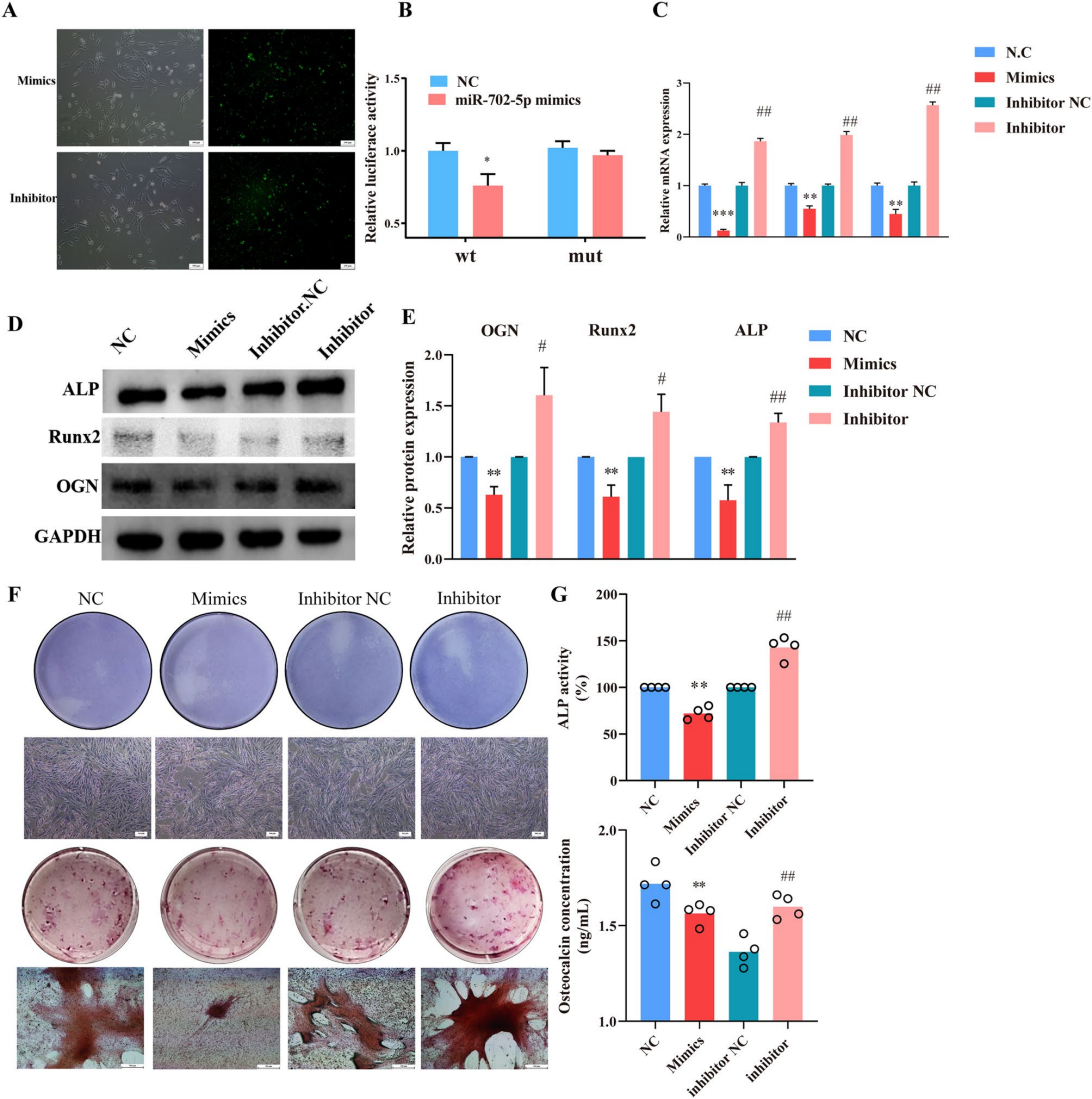
MiRNA-702-5p targeted OGN inhibit the osteoblast’s activity. (**A**) The transfection efficiency of mimics and inhibitor in ROBs was approximately higher than 80%. (**B**) Interaction between miRNA-702-5p and OGN 3’-UTR tested in the luciferase reporter assays. *P < 0.05, compared to the N.C groups. (**C**–**E**) Overexpression of miRNA-702-5p decreases the mRNA and protein levels of OGN and Rinx2. In contrast, inhibition of miRNA-702-5p effectively reverses the situation. **P < 0.01, **P < 0.001, ***P < 0.0001, compared to the N.C groups, #*P* < 0.05, ##*P* < 0.01 compared to the inhibitor N.C groups. (**F**) Overexpression of miRNA-702-5p inhibited ALP positive staining, and alizarin red staining. (**G**) ALP activity (%) and osteocalcin concentrations (ng/mL) results compared with negative control and inhibition of miRNA-702-5p showed the opposite effect. **P < 0.001, compared to the N.C groups, ##*P* < 0.01 compared to the inhibitor N.C groups.

To validate the relationship between miR-702-5p and OGN in ROBs, overexpression and knockdown of these miRNAs were used to study their functional roles. We transfected ROBs with 50 μM miRNA-702-5p mimics, 100 μM miRNA-702-5p inhibitor into ROBs for 16 h, two other groups of cells were treated with the same concentrations of NC and inhibitor NC. mRNA and protein levels were assessed by qPCR and Western blotting. As a result of transfecting cells with miR-702-5p mimic to overexpress miR-702-5p, OGN and Runx2 expression was reduced at the mRNA and protein levels when compared to cells treated with miR mimic NC. Besides, miR-702-5p overexpression reduced Runx2 mRNA and protein levels. Anti-miRs knocked down miR-702-5p expression (Fig. 3A–C), consequently, Runx2 and OGN mRNA and protein levels increased significantly (Fig. 3D, E). The expression of OGN was increased by the repression of miR-702-5p when compared to cells treated with anti-miR NC. In addition, miR-702-5p mimic treatment could efficiently decrease the activity of ALP and formation of calcified nodules, and miR-702-5p inhibitor treatment had oppositive effects (Fig. 3F, G).

Taken together, our results suggest that miR-702-5p targets OGN at mRNA and protein levels. Intermittent glucose stimulation promoted miR-702-5p, with a concomitant decrease in OGN and Runx2 expression. The experiments on the function of miRNA-702-5p showed that it can enhance the mineralization of osteogenic cells by targeting the OGN/Runx2 pathway.

### High glucose regulated OGN/Runx2 pathway in osteoblasts

**Figure 4:**
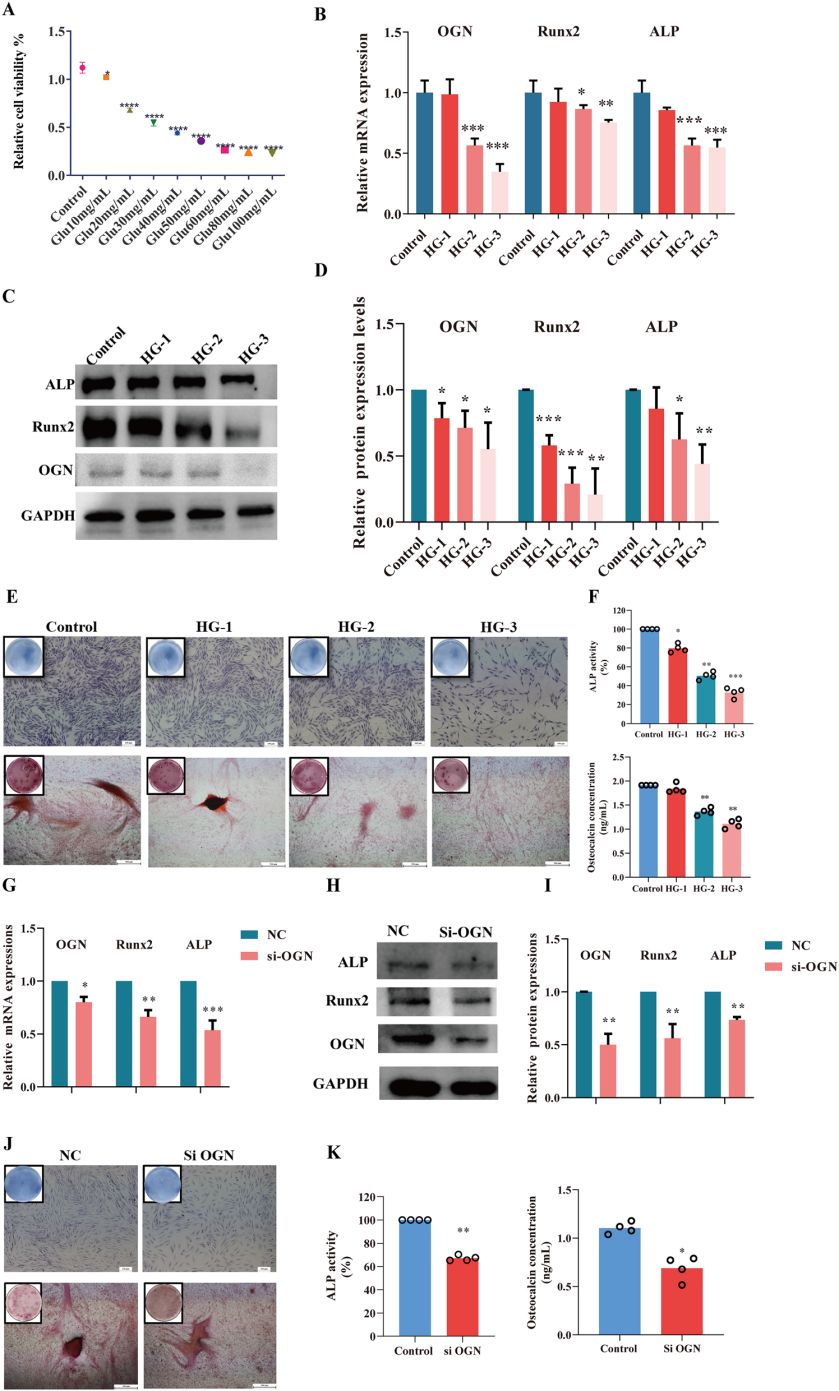
HG suppresses the proliferation and mineralization of osteoblasts by regulating OGN/Runx2 pathway. (**A**) ROBs were treated with different concentrations of glucose, the survival rate of osteoblasts was measured by MTT. (**B**) qRT-PCR was performed to analyze the expression levels of OGN, Runx2, ALP. (**C**, **D**) western blot analysis showed the protein levels of OGN, Runx2, and ALP. (**E**) ALP staining and alizarin red staining was used to determine the effect of HG on the proliferation and mineralization of osteoblasts. (**F**) ALP activities and osteocalcin concentrations (ng/mL) were calculated proliferation and mineralization of osteoblasts in control and HG groups. (**G**–**I**) The mRNA level and protein expressions of OGN, Runx2, ALP after transfection with NC, and Si-OGN for 24 h. (**J**) ALP staining and alizarin red staining after transfection with NC, and Si-OGN. (**K**) ALP activities and osteocalcin concentrations (ng/mL) were calculated after transfection with NC, and Si-OGN. Data depict the mean ± standard deviation (mean ± SD) and are representative of three independent experiments. *P < 0.05, **P < 0.01, ***P < 0.001 compared with the control groups.

As part of our study, we examined the effect of glucose on the expression of osteoblastic genes in osteoblasts. The ROBs were induced with different concentrations of glucose, and viability assay results showed that in the cell group treated with 10 mg/mL glucose, the growth viabilities were 91.17%, while the 50 mg/mL groups decreased to 31.97% at 24 h, which were different from the control groups (*P* < 0.01, Fig. 4 A). The relative cell viability dropped into a plateau when the glucose was higher than 50 mg/mL. Therefore, the concentrations of 10, 20, and 50 mg/mL were considered to be glucose treatments for analyzing the effect of high glucose on osteoblasts.

QRT-PCR results suggested that the gradient concentration of glucose decreased the mRNA expressions of OGN, Runx2, and ALP (*P* < 0.05, *P* < 0.01, *P* < 0.001, Fig. 4B). Western blotting results indicated that 20, 50 mg/mL glucose inhibited the protein expressions of OGN, Runx2 and ALP (*P* < 0.05, *P* < 0.01, *P* < 0.001, Fig. 4C, D). In addition, the activity of ALP and formation of calciferated nodules in ROBs and the concentration of osteocalcin were decreased by treatment with 20 or 50 mg/mL glucose compared with those in the control group. (Fig. 4E, F). To examine the expression of OGN downstream gene, we transfected ROBs with Si-OGN. In brief, Si-OGN could decrease the mRNA expressions and protein levels of OGN, Runx2 and ALP (*P* < 0.05, *P* < 0.01, *P* < 0.001, Fig. 4 G, H, I), and depressed osteoblast ALP activity and mineralization finally (Fig. 4J, K). These results suggested that HG could inhibit cell proliferation and mineralization in a dose-dependent manner.

### OGN and Runx2 was down-regulated in DOP models

**Figure 5:**
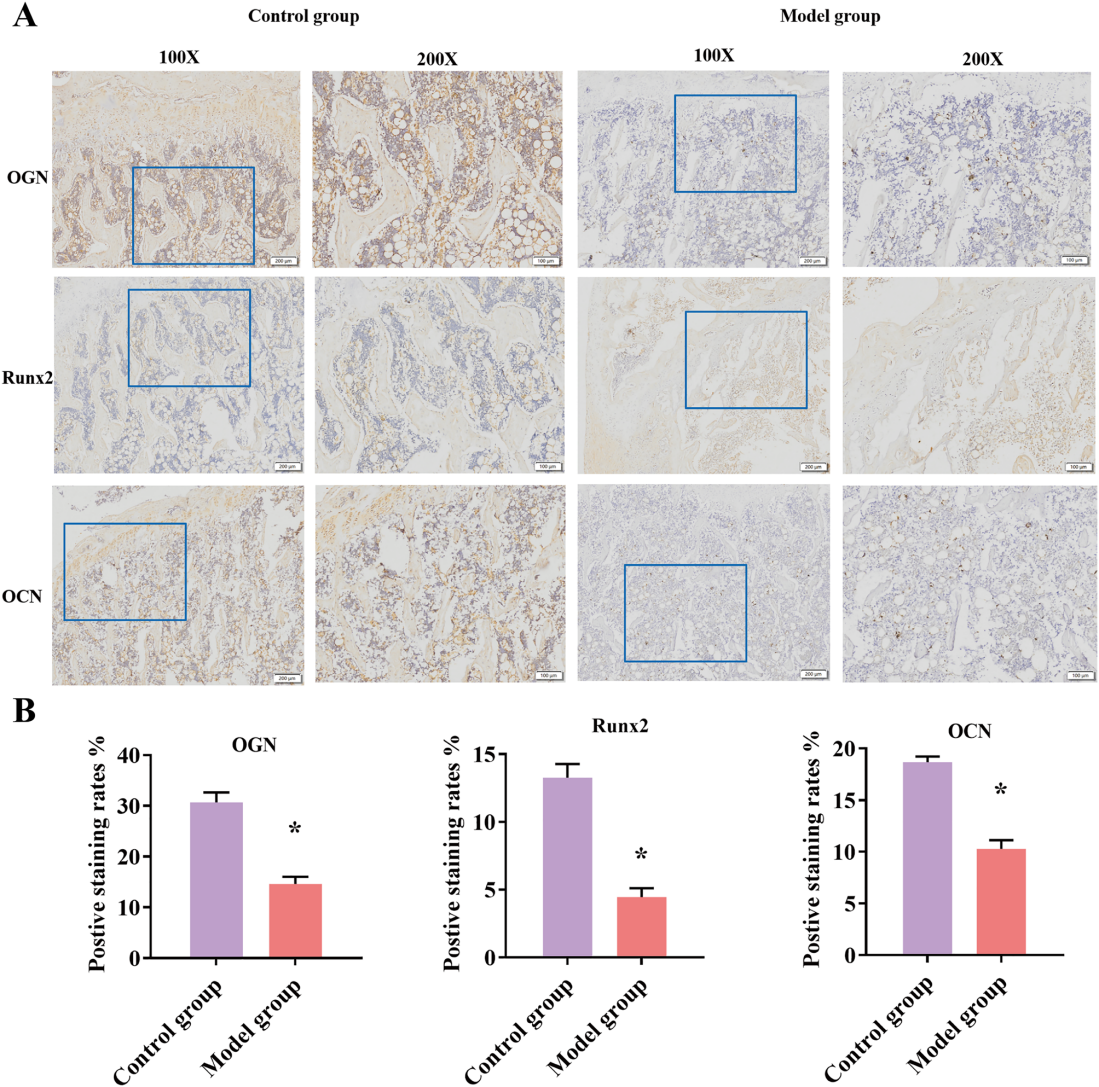
OGN, Runx2 and OCN positive expression rates were down-regulated in the DOP models. (**A**) Representative immunohistochemistry (IHC) image of OGN, Runx2 and OCN staining of femur with DOP and control groups (× 100, scale bar: 200 μm, × 200, scale bar: 100 μm). (**B**) OGN, Runx2 and OCN positive expression rates of femur in each group. *P < 0.01, compared with control groups.

In order to verify the OGN/Runx2 pathway in the DOP models, we detected the activities of OGN, Runx2, and OCN in the femurs to further characterize the crucial molecular changes in the bone metabolism of diabetes by detecting the immunohistochemistry staining of OGN, Runx2, and OCN. Quantification results immunohistochemistry staining showed that OGN and Runx2 expressions were markedly decreased in rats of the DOP groups (*P* < 0.01, Fig. 5A, B).

## Discussion

Osteoporosis can be divided into primary and secondary osteoporosis, and DOP is currently considered a prevalent type of secondary osteoporosis^19^. During the progression of DOP, an abundance of glucolipid metabolites builds up in the bone tissue, causing a reconfiguration of the metabolism of bone cells. The deleterious effects of the diabetic microenvironment on bone strength and bone mass have recently received increased acknowledgement^20^. Nevertheless, the complete understanding of the underlying process of bone loss in the diabetic microenvironment remains unclear, and there is currently no viable treatment approach for DOP. In our work, animals were subjected to a high-sugar, high-fat diet to induce insulin resistance, followed by intraperitoneal injection of small doses of STZ to induce mild damage to islet beta cells, leading to compensatory secretion abnormalities of insulin and subsequent development of hyperglycemia. This modeling approach has been corroborated by previous studies^21,22^. Our findings align with prior research, as blood glucose levels exceeded 16.7 mmol/L for a duration of 2 weeks in our investigation. Subsequently, we examined bone microarchitecture, and found that T2DM induced the decrease of bone microarchitecture parameters BV/TV, Tb.Th, and BMD. Besides, the femoral trabecula was broken and the number of trabeculae was decreased in the T2DM rats. These data suggested that T2DM successfully induces the lower bone mass of rats. Our study results were similar to those of previous studies, which showed a lower BMD, bone volume, and bone histomorphometry in T2DM rats^3,23^. We hypothesized that T2DM can regulate bone related gene and protein expression and disturb the dynamic balance of bone formation and resorption.

**Figure 6:**
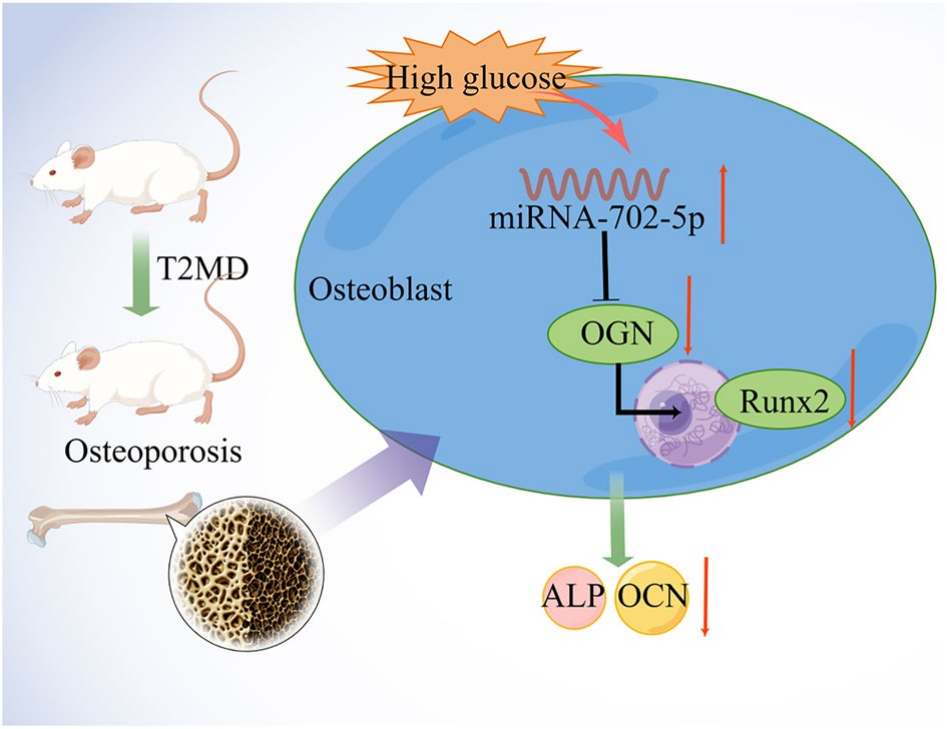
The diagram of miR-702-5p/OGN signaling pathway in DOP.

Osteoblasts and osteoclasts interact dynamically to repair and remodel bones. Conversely, bone diseases may result from an imbalance between osteoblasts and osteoclasts. High glucose could inhibit the proliferation and differentiation of osteoblasts^24^. However, the specific mechanisms behind the altered expression and transcription of genes in osteoblasts induced by high glucose, leading to biological processes, remain unknown. Thus, understanding of the transcriptome signature and specific molecular mechanisms of high glucose injury is necessary to provide the strategies for their prevention and treatment. MiRNAs are considered the mediated factors regulating osteoblasts' proliferation and function or osteoclasts^25,26^. In this research, miRNA-seq was carried out to explore the miRNA characteristic in the HG treated ROBs. We concluded 10 miRNAs were differential expressed between these two groups. MiR-702-5p was significantly up-regulated by glucose treatment in the RT-PCR verification experiments, which indicated the biological function in inhibiting osteogenic differentiation. In previous study, miR-702-5p was proved to regulate diabetic encephalopathy in db/db mice^27^. Indeed, there were other miRNAs were significantly differential expressed, such as miR-330-5p, miR-185-5p, miR-190a-3p, however, the miR-702-5p was most significantly differential expressed. Also, the other differential expressed miRNAs will be verified in our further studies. ROBs responded to intermittent glucose stimulation by promotion of miR-702-5p, with a concomitant decrease in a target gene for these miRNAs. It's no coincidence that miR-702 was verified to impair osteogenesis by suppressing signaling pathway in the previous study^28^. Osteocalcin gene, such as Runx2, OGN is known to be involved in bone remodeling, was downregulated by glucose stimulation. This study offers a new insight into the action of miRNAs and consequential gene regulation after being disrupted by high levels of glucose, which affects the differentiation and mineralization of osteoblasts (Fig. 6). Based on the results of our study, the downregulation of miR-702-5p has been shown to enhance osteoblast cell differentiation and increase ALP activity and the expression of osteogenic genes by targeting OGN, thereby elucidating the mechanism underlying high glucose-induced osteoblast dysfunction. It is postulated that miR-702-5p may play a pivotal role in the regulation of bone homeostasis under both normal physiological conditions and in cases of DOP. However, further research is necessary to validate the impact of miR-702-5p in vivo, specifically through the targeted deletion of miR-702-5p in osteoblasts.

Glucose generally has effect on bone in vivo, especially in the diabetic osteoporosis. Osteoporosis is caused clinically by the osteopenic agent glucose, which accelerates osteoblast death and inhibits bone remodeling genes^29^. OGN is a small leucine-rich proteoglycan family member which enhances bone formation parameters in osteoblasts, which is positively correlated with phenotype and mineralization of osteoblasts^30^. The previous studies suggest that higher serum OGN levels is a risk factor associated with decreased BMD and vertebral fractures in T2DM postmenopausal women. Moreover, clinical studies link osteoglycin to insulin resistance and other diseases^31^. OGN may be a novel marker of a muscle-pancreatic-bone axis^32^. Prior research has suggested a correlation between OGN and the regulation of glucose metabolism in response to alterations in energy homeostasis, a connection that is further supported by studies conducted on diabetic osteoporotic patients and diabetic osteoporotic rats^33,34^. In glucose tolerance test on mice, it was observed that treatment with OGN led to a dose-dependent reduction in blood glucose levels. Additionally, in vitro treatment with OGN resulted in a dose-dependent upregulation of Ins1 and Ins2 mRNA expression, which are associated with insulin secretion. Furthermore, OGN treatment was found to enhance the decrease in glucose levels in response to insulin, indicating a potential enhancement of insulin action by OGN. These findings suggest that OGN may possess metabolic activity^35^. Morever, osteoblast serves as a crucial target for insulin in regulating systemic glucose balance, and highlights bone resorption as the primary mechanism governing osteocalcin activation^36^. A decrease in OGN expression, which further results in a decrease of the expression of osteogenesis-related factors^37^. In previous studies, glucose could decrease the expression of OGN, for instance, advanced glycation end products (AGE) decrease OGN expression in myoblastic cells^36^. Thus, we speculated OGN was high correlation with DOP. From the trabecular bone histological results, DOP markedly decreased OGN and Runx2 activities, which indicated OGN has an effect on osteogenesis-related factors, and might play an important role in bone formation. In previous studies, OGN played a significant role in osteoporosis, which is characterized by altered differentiation of BMSCs into osteoblasts and adipocytes. OGN could have a significant impact on the deceleration of osteogenesis specific genes, such as RUNX2, OCN, ALP and Wnt5b^38^. This study concluded that high glucose suppresses osteoblasts’ proliferation and mineralization by regulating the OGN/Runx2 pathway.

Collectively, the action of miRNA-702-5p and target gene OGN regulation follow glucose-induced osteoblast differentiation perturbation. We observed substantial differences in the expression of target genes between diabetic osteoporosis model rats and control rats, suggesting a potential role of a subset of miRNAs in the regulation of bone remodeling and osteoblast differentiation by glucose.

### Supplementary Information

Supplementary Information.

Supplementary Table 1.

## Data Availability

The original contributions presented in the study are included in the article/Supplementary Material. Further inquiries can be directed to the corresponding author.

## Abbreviations

*ALP*: Alkaline phosphatase
*BMD*: Bone mineral density
*BS/BV*: Bone surface density
*BV/TV*: Bone volume fraction
*DOP*: Diabetic osteoporosis
*H&E*: Hematoxylin and eosin
*IF*: Immunofluorescence
*IHC*: Immunohistochemistry
*micro-CT*: Micro-computed tomography
*OCN*: Osteocalcin
*OGN*: Osteoglycin
*PBS*: Phosphate buffered saline
*ROBs*: Rat calvarial osteoblasts
*Runx2*: Runt-related transcription factor 2
*STZ*: Streptozotocin
*T2DM*: Type 2 diabetes mellitus
*Tb.Th*: Trabecular thickness
