# Supplementary material for: miRNA-seq analysis of high glucose induced osteoblasts provides insight into the mechanism underlying diabetic osteoporosis
Source: Sci Rep. 2024 Jun 11;14:13441. doi: 10.1038/s41598-024-64391-z (PMC11166950; doi:10.1038/s41598-024-64391-z)
Supplement: Supplementary file 1 — Supplementary Information. [file 41598_2024_64391_MOESM1_ESM.pdf]

Figure 3 D

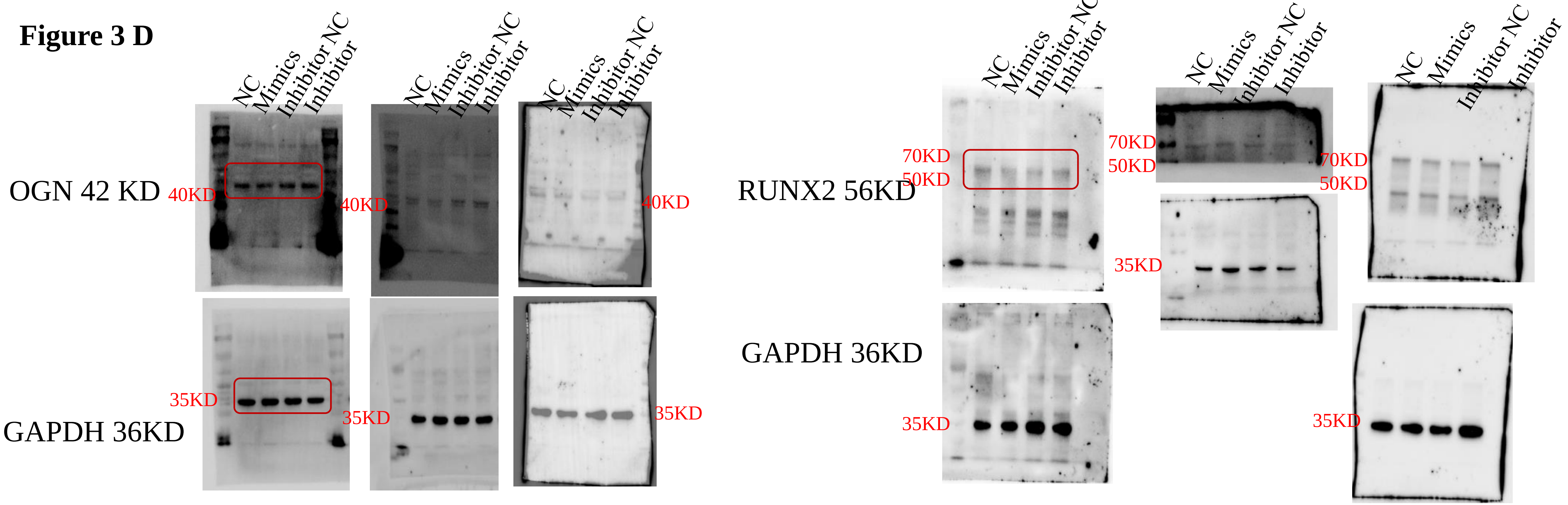

NC
Mimics
Inhibitor NC
Inhibitor
OGN 42 KD
40KD
GAPDH 36KD
35KD
RUNX2 56KD
70KD
50KD
35KD
GAPDH 36KD
35KD

Figure 3 D

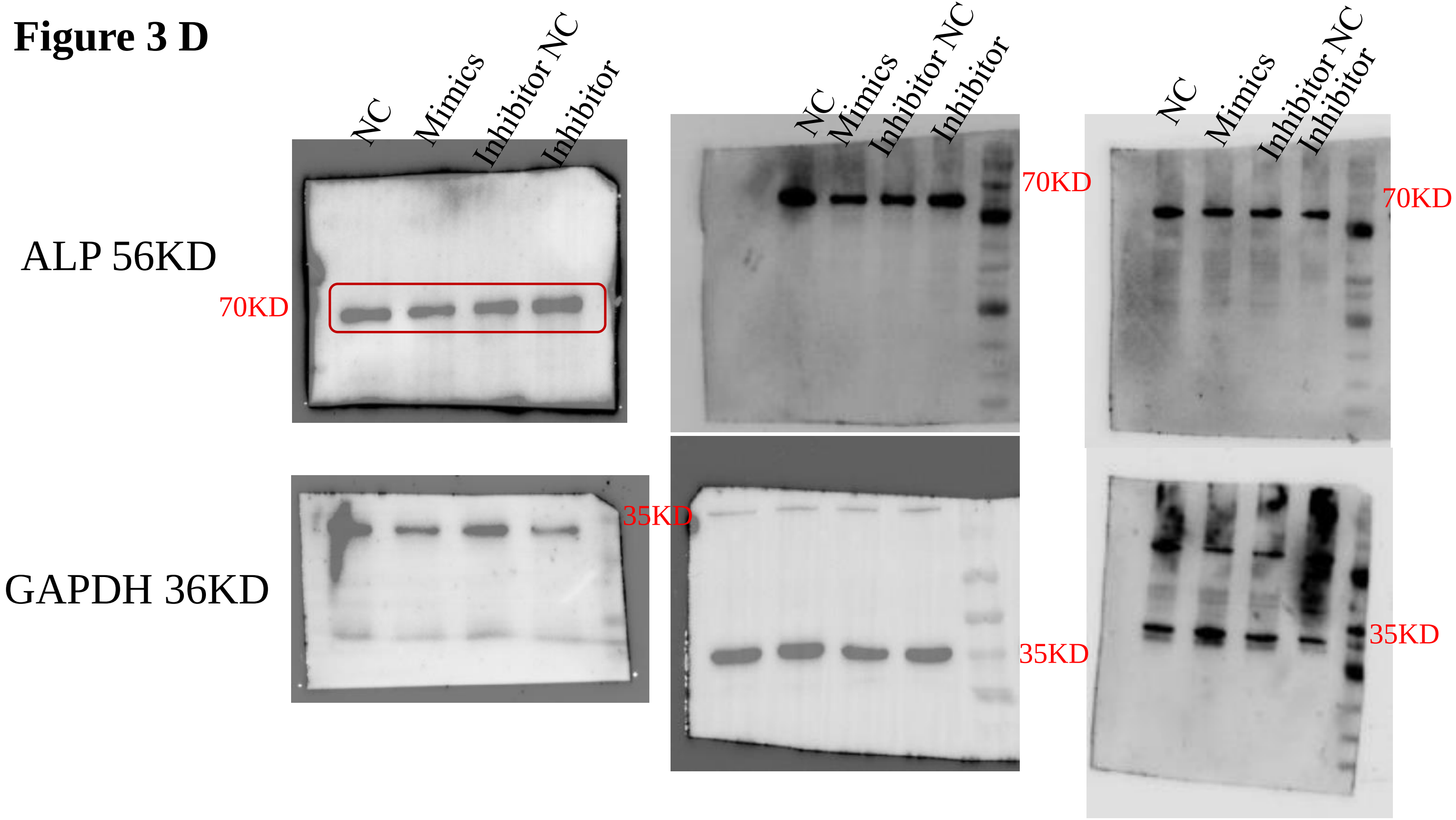
ALP 56KD
GAPDH 36KD
NC
Mimics
Inhibitor NC
Inhibitor
70KD
35KD
NC
Mimics
Inhibitor NC
Inhibitor
70KD
35KD
NC
Mimics
Inhibitor NC
Inhibitor
70KD
35KD

Figure 4 C

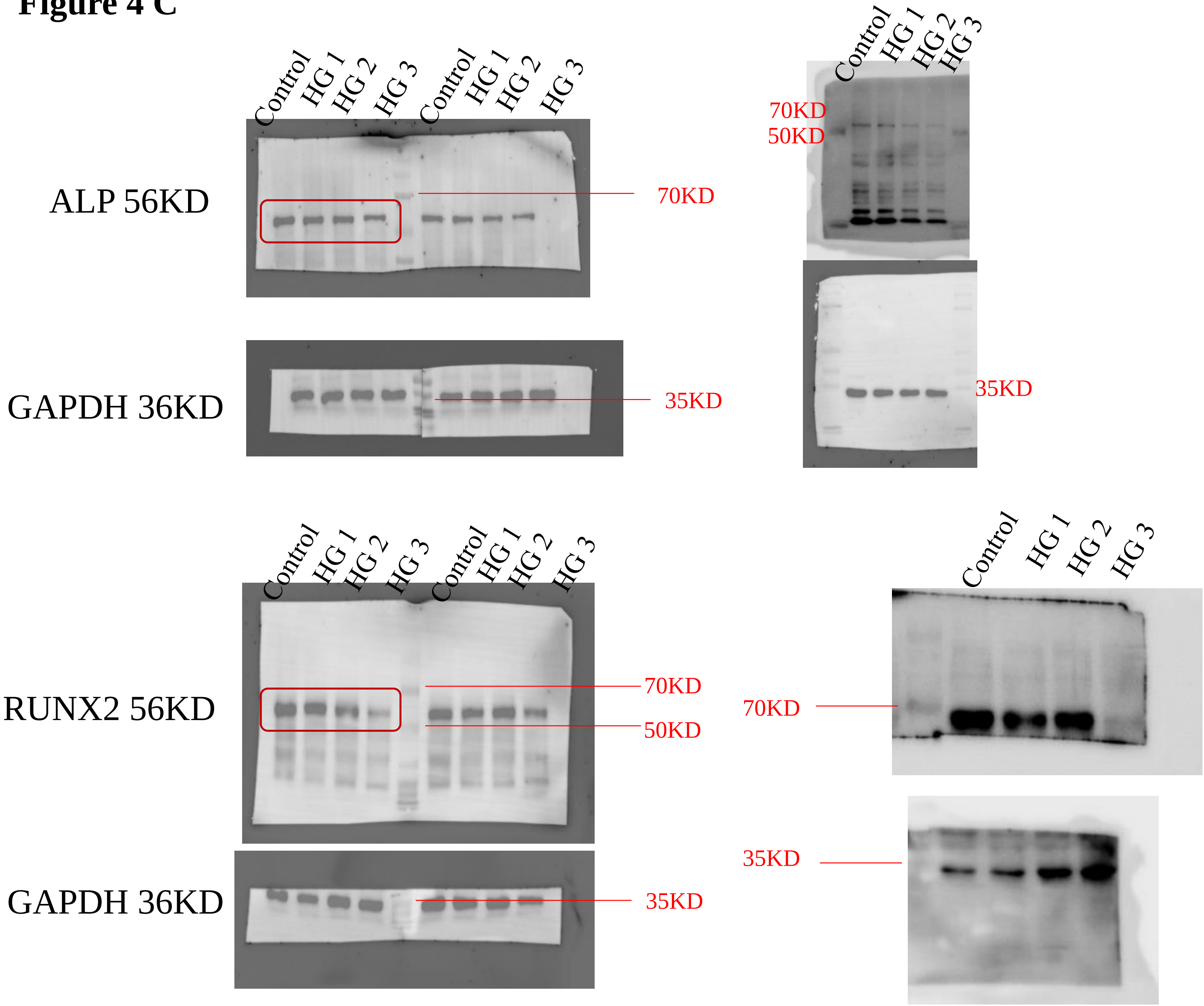

Control
HG 1
HG 2
HG 3
Control
HG 1
HG 2
HG 3
ALP 56KD
70KD
GAPDH 36KD
35KD
70KD
50KD
35KD
RUNX2 56KD
70KD
50KD
GAPDH 36KD
35KD
70KD
35KD

Figure 4 C

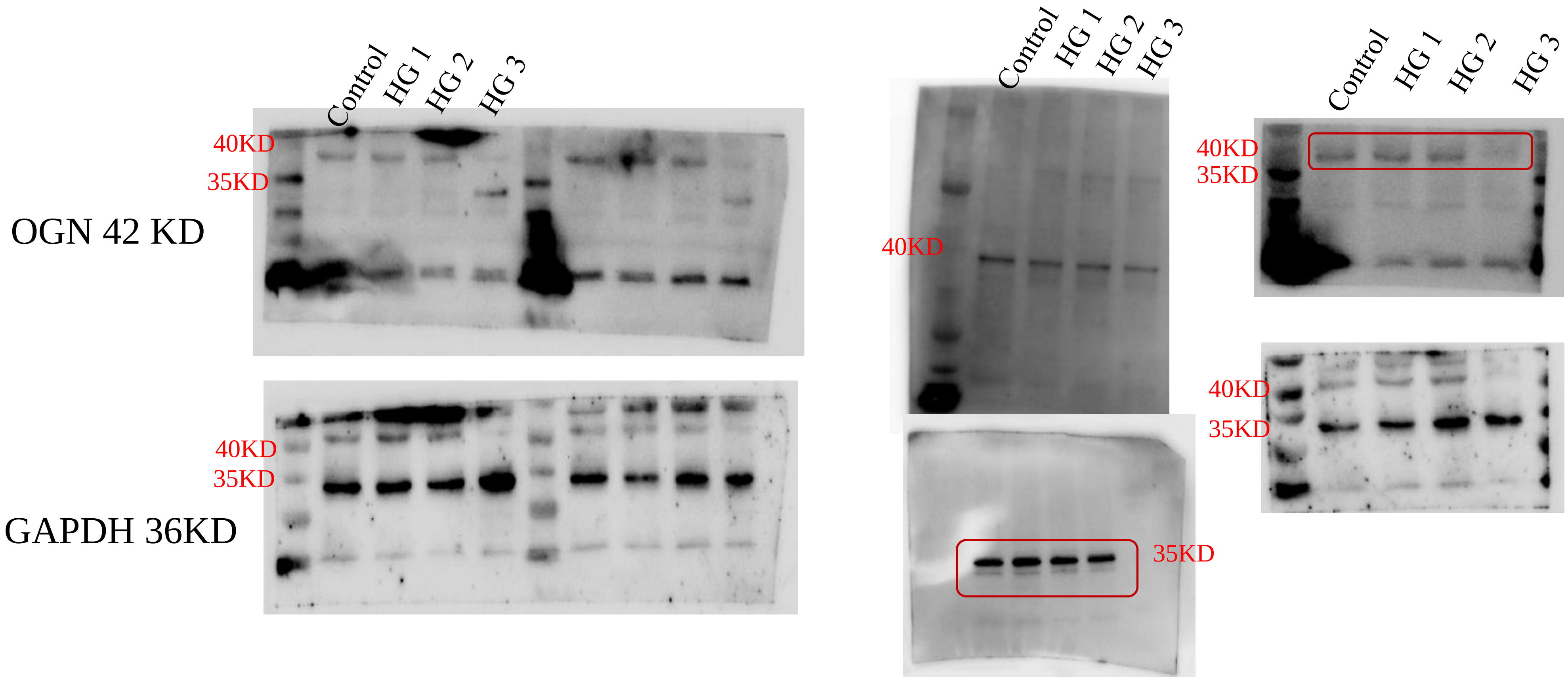

Control
HG 1
HG 2
HG 3
40KD
35KD
OGN 42 KD
40KD
35KD
GAPDH 36KD
Control
HG 1
HG 2
HG 3
40KD
35KD
Control
HG 1
HG 2
HG 3
40KD
35KD
40KD
35KD

Figure 4 H

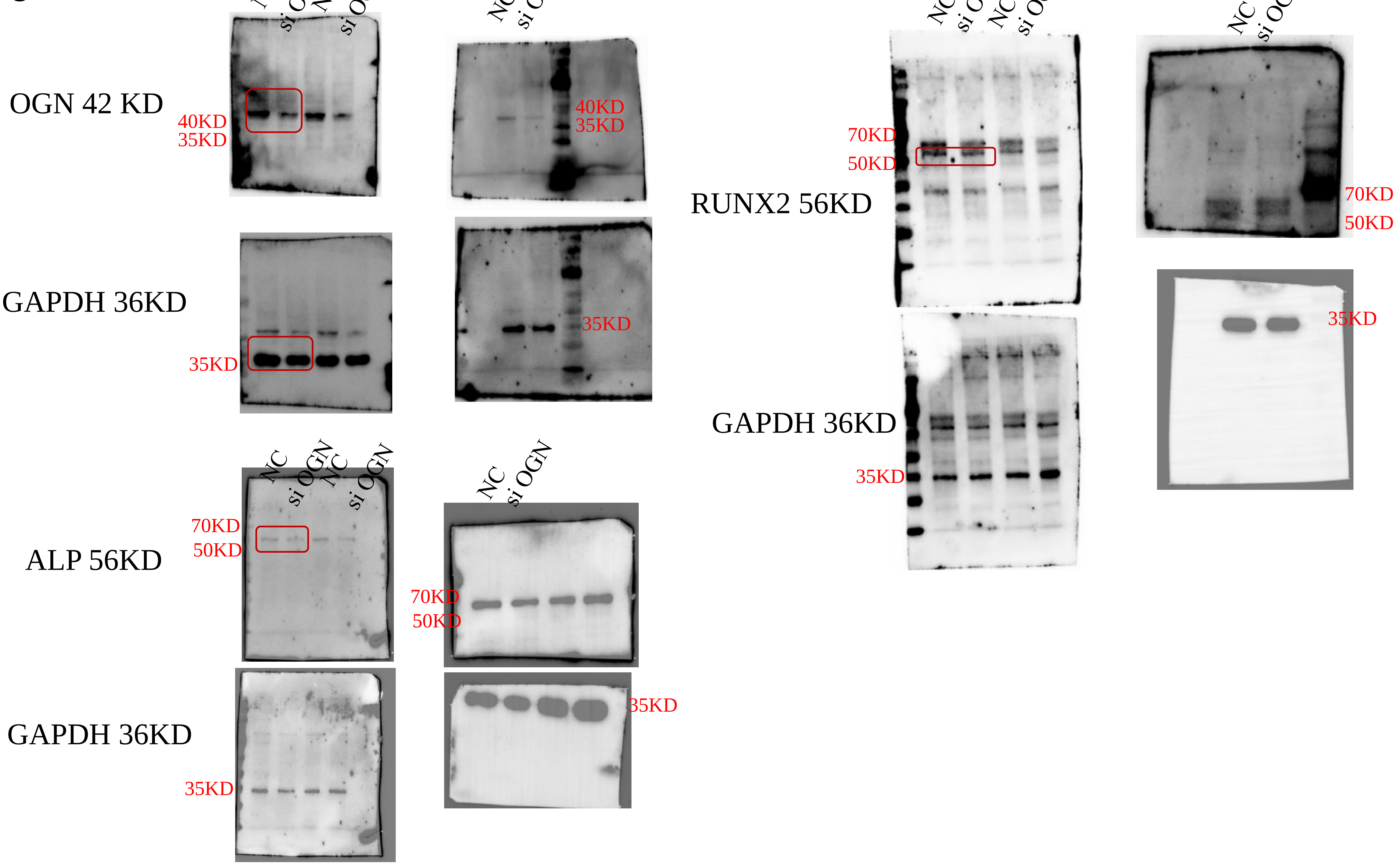

NC
si OGN
NC
si OGN
OGN 42 KD
40KD
35KD
GAPDH 36KD
35KD
ALP 56KD
70KD
50KD
GAPDH 36KD
35KD
RUNX2 56KD
70KD
50KD
GAPDH 36KD
35KD
