# Supplementary material for: miRNA-seq analysis of high glucose induced osteoblasts provides insight into the mechanism underlying diabetic osteoporosis
Source: Sci Rep. 2024 Jun 11;14:13441. doi: 10.1038/s41598-024-64391-z (PMC11166950; doi:10.1038/s41598-024-64391-z)
Supplement: Supplementary file 2 — Supplementary Table 1. [file 41598_2024_64391_MOESM2_ESM.docx]

Supplementary Table 1 Sequences of primers used in qRT-PCR

| Gene name | Former Sequencing | Reverse Sequencing |
| --- | --- | --- |
| miR-702-5p | GCGGTGAGTGGGGTGGTT | AGTGCAGGGTCCGAGGTATT |
| U6 | CGTTCGTGAAGCGTTCCA | AGTGCAGGGTCCGAGGTATT |
| OGN | AAGTGCTCCCAGTGACCTTCCC | TGTGGCTCCCCTTTCCTTAGGC |
| Runx2 | CATGGCCGGGAATGATGAG | TGTGAAGACCGTTATGGTCAAAGTG |
| ALP | CACGGCGTCCATGAGCAGAAC | CAGGCACAGTGGTCAAGGTTGG |
| β-actin | GGAGATTACTGCCCTGCTCCTA | GACTCATCGTACTCCTGCTTGCTG |
